# Increased Percentages of T Helper Cells Producing IL-17 and Monocytes Expressing Markers of Alternative Activation in Patients with Sepsis

**DOI:** 10.1371/journal.pone.0037393

**Published:** 2012-05-31

**Authors:** Milena Karina Colo Brunialti, Michelle Carolina Santos, Otelo Rigato, Flavia Ribeiro Machado, Eliezer Silva, Reinaldo Salomao

**Affiliations:** 1 Division of Infectious Diseases, Department of Medicine, Hospital Sao Paulo, Escola Paulista de Medicina, Universidade Federal de Sao Paulo, Sao Paulo, Brazil; 2 Critical Care Unit, Hospital Sirio-Libanes, Sao Paulo, Brazil; 3 Division of Anesthesiology, Department of Surgery, Hospital Sao Paulo, Escola Paulista de Medicina, Universidade Federal de Sao Paulo, Sao Paulo, Brazil; 4 Critical Care Unit, Hospital Albert Einstein, Sao Paulo, Brazil; University of Sao Paulo, Brazil

## Abstract

**Background:**

A shift from Th1 to Th2 as well as an increase in Treg CD4+T cell subsets has been reported in septic patients (SP). Furthermore, these patients display modulation of monocyte function, with reduced production of pro-inflammatory cytokines upon LPS stimulus, which resembles the phenotype of alternatively activated macrophages. In this study, we evaluated the percentages of T cells differentiated into Th1, Th17 and Treg subsets, as well as the percentage of monocytes expressing markers of alternatively activated monocytes/macrophages (AAM) in SP.

**Methodology/Principal Findings:**

Peripheral blood mononuclear cells (PBMC) were obtained from 32 healthy volunteers (HV) and from SP at admission (D0, n = 67) and after 7 days of therapy (D7, n = 33). Th1 and Th17 (CD3+CD8−) lymphocytes were identified by the intracellular detection of IFN-γ and IL-17, respectively, spontaneously and after PMA/Io stimulation, and Treg cells were identified by Foxp3+CD127- expression. Monocytes were evaluated for CD206 and CD163 expression. Absolute numbers of CD4+T lymphocytes were measured in whole blood samples by flow cytometry. The Mann-Whitney or Wilcoxon test was applied, as appropriate. The percentage of Th1 cells was lower in SP than in HV at admission after PMA/Io stimulation, whereas the percentage of Th17 cells was higher. In patients’ follow-up samples, a higher percentage of Th1 cells and a lower percentage of Th17 cells were observed on D7 compared with the D0 samples. Treg cells remained unchanged. Septic patients showed a markedly increased proportion of monocytes expressing CD163 and CD206.

**Conclusions/Significance:**

Upon *in vitro* stimulus, the percentage of T helper lymphocytes producing IL-17 was higher in SP than in HV at admission, and the percentage producing IFN-γ was lower, a pattern that was reversed during follow-up. The increased expression of CD163 and CD206 indicates that monocytes may acquire the AAM phenotype during sepsis.

## Introduction

Inflammatory responses are modulated during sepsis, and different components of inflammation are upregulated or downregulated, depending on the type of cell and its function [1]. It has been suggested that a state of hyporesponse or immune paralysis follows the initial inflammatory response in humans and experimental sepsis, which is related to susceptibility to nosocomial infections and late lethality [2]–[5].

A reduction in lymphocyte populations and increased apoptosis have been observed during sepsis, with a shift from a T helper (Th)1 to a Th2 pattern of cytokine expression [2]. Two other CD4+ T lymphocyte subpopulations, regulatory T cells (Treg) and Th17 cells, have been characterized and found to have major antagonistic effects on the inflammatory response, which may play a role in sepsis [6].

Tregs suppress the activity of lymphocytes and macrophages [7]. Increased proportions of Tregs have been described in human and experimental sepsis [8]–[10], yet their role in sepsis-induced immune suppression has not been elucidated [11]–[13]. Th17 cells have recently been characterized as a distinct CD4+ T subset [14]. Naïve CD4+ T cells will differentiate into Th17 cells in the presence of IL-6 and transforming growth factor (TGF) -β [6], [15], [16]. Different pathogens may trigger the induction of IL-17, and protective and detrimental roles for IL-17 have been shown in experimental infections; however, information on IL-17 in clinical sepsis is scarce.

The distinct CD4+ T cell subsets, among other cell types, play major roles during the differentiation of macrophages into distinct phenotypes. IFN-γ induces classical macrophage activation, leading to enhanced microbicidal or tumoricidal activity and the production of high levels of pro-inflammatory cytokines as well as reactive oxygen and nitrogen species. IL-4 and IL-13 induce alternative activation, resulting in increased arginase activity (repair activity) and enhanced mannose receptor expression. IL-10, among other stimuli, induces a more profound monocyte deactivation, which leads to the production of high levels of IL-10 and low levels of IL-12 [17], [18]. IL-4/IL-13- and IL-10-induced macrophages are referred to as alternatively activated macrophages (AAM), or more precisely as wound-healing and regulatory macrophages, respectively [18]. Interestingly, IL-4 and or IL-13 induce AAM that express CD206 [19], while IL-10 induces AAM that express CD163 on the cell surface [20]. IL-21, one of the cytokines related to Th17 cells, is implicated in Th2 effector function and alternative macrophage differentiation [21]. Recent evidence indicates that Tregs induce an AAM phenotype, along with the expression of both receptors, increased phagocytosis and suppressed production of pro-inflammatory cytokines upon LPS stimulation [22].

Monocyte functions have been shown to be modulated during sepsis [23]–[28]. Decreased expression of CD14 and HLA-DR on the surface of monocytes [23]–[25] and low production of inflammatory cytokines by peripheral blood cells have been found in septic patients [3], [4], [26]. Interestingly, monocytes that were hyporesponsive with respect to the production of inflammatory cytokines [24] showed preserved reactive oxygen species generation [27]. It is conceivable that these functional changes may indicate the differentiation of monocytes into the phenotypes of alternatively activated cells.

Given that the interplay between T lymphocyte subpopulations and monocytes/macrophages may have a role in the pathophysiology of sepsis, we evaluated T helper differentiation into Th1, Th17 and Treg cells and the percentage of monocytes expressing CD206 and CD163, which are markers of AAM, in septic patients.

## Materials and Methods

### Patients and Healthy Volunteers

This study was approved by the ethics committees of the participating hospitals, Sao Paulo Hospital (Study number 1477/06), Albert Einstein Hospital (Study number 07/549) and Sirio Libanes Hospital (Study number 2006/27). All participants were enrolled after informed consent was obtained. Patients admitted to the Intensive Care Units from the 3 hospitals, localized in Sao Paulo, Brazil, were enrolled with clinical diagnoses of sepsis according to the ACCP/SCCM consensus conference [29]. Samples were obtained from 67 septic patients at admission (D0). Thirty-three of the patients had a second sample collected after 7 days (D7) of therapy. Clinical and epidemiological data were obtained from patients during their stay in the Intensive Care Units. The outcomes are reported as in-hospital mortality. An attempt to match age and gender was made using 32 healthy volunteers with similar characteristics to the septic patients.

### Peripheral Blood Mononuclear Cell (PBMC) Isolation, Cryopreservation and Thawing

Fifty milliliters of blood was collected in sodium heparin-treated tubes (BD Biosciences, Franklin Lakes, NJ, USA). Peripheral blood mononuclear cells were obtained using the Ficoll gradient method (Ficoll-Paque PLUS; GE Healthcare Bio-Sciences AB, Uppsala, Sweden), frozen in fetal bovine serum (Invitrogen-Gibco, Gaithersburg, MD, USA) with 10% dimethyl sulfoxide (Calbiochem, La Jolla, CA, USA) and stored in liquid nitrogen until use. Upon thawing, the cells were washed and suspended in R10 (RPMI 1640 medium supplemented with 10% fetal bovine serum, 1% HEPES buffer solution 100 mM, 1% L-glutamine 200 mM, 1% sodium pyruvate 100 mM, 1% penicillin/streptomycin 100×, and 0.1% 2-mercaptoethanol 55 mM, all from Invitrogen). The standard cell concentration was 1×10^6^ cells/mL.

### Intracellular Staining for IL-17A and IFN-γ

PBMCs were stimulated with 20 ng/mL phorbol 12-myristate 13-acetate (PMA) and 0.5 µL 1 mM ionomycin (Io) (Sigma, Saint Louis, MO, USA) at 37°C and under 5% CO_2_ for 30 minutes. Brefeldin A (1 µg/mL; Sigma) was added, and the samples were incubated for an additional 15 h. PMA/ionomycin triggers a strong production of cytokines *in vitro* and is largely used to evaluate intracellular detection of cytokines from different T lymphocyte subpopulations [30].

**Figure 1:**
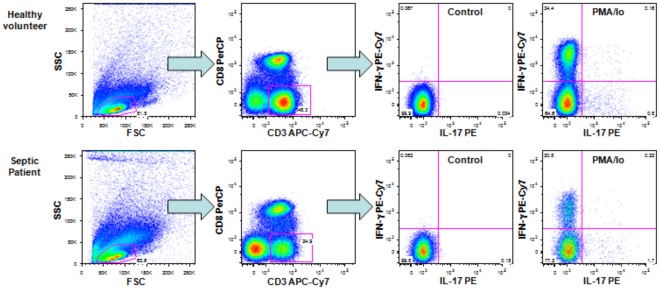
Strategy for the analysis of Th1 and Th17 lymphocytes. Dot plots shown are representative of one healthy volunteer and one septic patient. T cells were identified based on CD3-APC-Cy7 staining and forward scatter versus side scatter parameters. For the analyses, T helper cells were gated as CD3+CD8−, and the percentages of cells producing IFN-γ or IL-17 were determined with the quadrants established based on the control samples.

The cells were stained with CD3-allophycocyanin-cyanine 7 conjugate (APC-Cy7) and CD8-perididin chlorophyll protein (PerCP) (clones SK7 and SK1, respectively, both from BD Biosciences) to identify the T helper lymphocyte population (CD3+CD8-) [30]. After washing with FACS buffer (0.15 M PBS (8.0 g NaCl, 0.2 g KH_2_PO_4_, 1.15 g Na_2_HPO_4_, 0.2 g KCl, distilled water to obtain 1 L, pH 7.2), 0.1% BSA (Sigma) and 2.0 mM EDTA (Sigma)), the samples were fixed and permeabilized by adding 750 µL 2× lysis solution (BD Biosciences) with 0.05% Tween 20 (Casa Americana de Artigos para Laboratorio, Sao Paulo, SP, Brazil) in the dark for 10 minutes. After centrifugation, the supernatant was discarded, and the cells were intracellularly stained to detect IL-17 and IFN-γ, using IL-17A-phycoerythrin (PE, clone eBio64CAP17, *e*Biosciences, San Diego, CA, USA) and IFN-γ phycoerythrin-cyanine 7-conjugated (PE-Cy7, clone B27, BD Biosciences) for 30 minutes in the dark. The samples were washed with FACS buffer and resuspended in fixation buffer (PBS supplemented with 1% paraformaldehyde; Polysciences, Warrington, PA, USA). T cells were identified based on CD3-APC-Cy7 staining and forward scatter versus side scatter parameters. In total, 70,000 events were acquired. CD4 T cells were gated as CD3+CD8−, and the percentages of cells producing IFN-γ or IL-17 were determined using thresholds based on unstimulated samples. Cells producing IFN-γ or IL-17 were considered for Th1 or Th17 cell characterization (Figure 1).

### T Regulatory Cell Immunophenotyping

**Figure 2:**
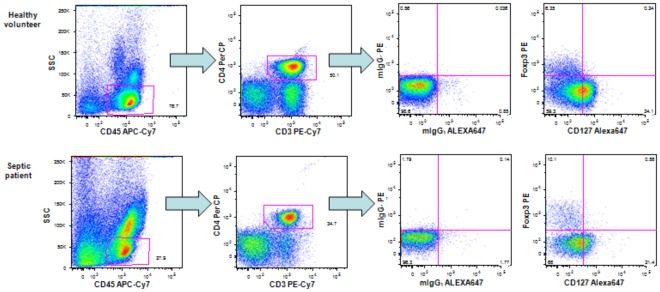
Strategy for the analysis of T regulatory lymphocytes. Dot plots shown are representative of one healthy volunteer and one septic patient. Fifty thousand events were acquired with CD3+ gating and forward scatter versus side scatter parameters. For the analyses, the first gate was determined based on low SSC and high CD45 APC-Cy7, and the second gate was based on CD4+CD3+ cells. The cells with the phenotype CD45+CD3+CD4+CD127-FOXP3+ were considered to be Treg lymphocytes. Quadrants or gates were established based on the isotype controls.

PBMCs were surface stained with CD45-APC-Cy7 (clone 2D1), CD3-PE-Cy7 (clone SK7), CD4-PerCP (clone SK3) and CD127 Alexa Fluor 647 (clone hIL-7R-M21) (all antibodies from BD Biosciences, San Jose, CA, USA) for 15 minutes at room temperature in the dark. The isotype mIgG_1_-Alexa Fluor 647 (clone MOPC-21 from BD Biosciences) was used as a control. After washing with FACS buffer, the cells were fixed, permeabilized and intracellularly stained with anti-forkhead box P3 (Foxp3)-PE antibody (clone 236A/E7) or isotype mIgG_1_-PE for 30 minutes at 4°C with buffers from an eBiosciences kit (San Diego, CA, USA). The samples were washed twice and resuspended in FACS buffer. Fifty thousand events were acquired from a gate based on CD3+ staining and forward scatter versus side scatter parameters. In analyses T helper lymphocytes were gated as CD45+CD3+CD4+ and Tregs characterized as CD127-Foxp3+ cells, as previously suggested [31], [32]. The analysis strategy is shown in Figure 2.

### Cell Surface CD163 and CD206 on Monocytes

**Figure 3:**
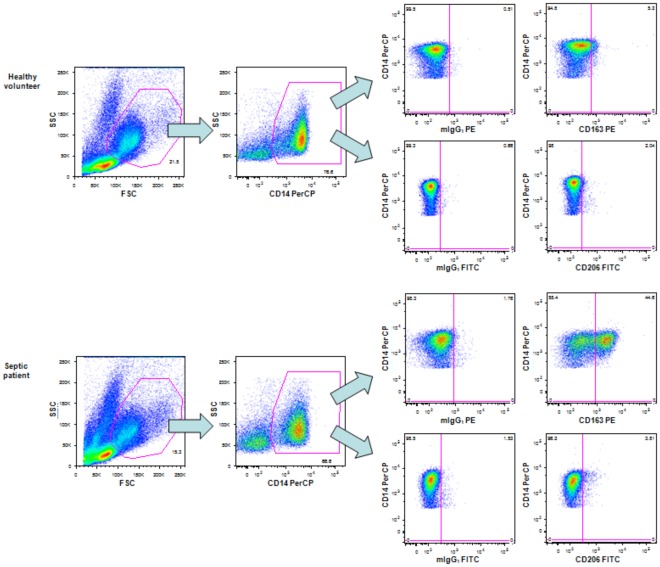
Strategy for the analysis of alternatively activated monocytes. Dot plots shown are representative of one healthy volunteer and one septic patient. Twenty thousand events were acquired based on forward scatter versus side scatter parameters and CD14-positive stained cells. The percentages of cells expressing CD163 or CD206 were determined with quadrants based on the isotype controls.

PBMCs were surface stained with CD14-PerCP (clone MφP9), CD16-APC-Cy7 (clone 3G8), CD163-PE (clone GHI/61) and CD206-fluorescein isothiocyanate (FITC, clone 19.2) (all antibodies from BD Biosciences) for 15 minutes at room temperature in the dark. The isotypes mIgG_1_-FITC and mIgG_1_-PE (both clone MOPC-21 from BD Biosciences) were used as controls for CD163 and CD206. After washing with FACS buffer, the cells were resuspended in fixation buffer. Twenty thousand events were acquired using a gate based on CD14-positive staining and forward scatter versus side scatter parameters, and the percentages of cells expressing CD163 or CD206 were determined using thresholds based on the isotype controls (Figure 3). Additional analysis of CD163 and CD206 expression was performed in monocytes subsets combining the expression of CD14/CD16 as follows: CD14++CD16−, CD14++CD16+ and CD14+CD16+ cells.

### Absolute CD4+T Lymphocyte Counting

Blood samples were collected in EDTA vacutainer tubes (BD Biosciences, Franklin Lakes, NJ, USA). Absolute numbers of CD4+T lymphocytes were counted using Multitest antibodies and TruCount tubes (BD Biosciences, San Jose, California, USA) according to the manufacturers’ instructions. Briefly, 50 µL of EDTA-treated whole blood was incubated for 15 minutes in the dark at room temperature with 20 µL CD3-FITC/CD8-PE/CD45-PerCP/CD4-allophycocyanin (APC, BD Biosciences). Erythrocytes were lysed with 450 µL lysing solution (BD Biosciences).

Absolute numbers of the T helper subsets (Th1, Th17 and Treg) for each individual were calculated based on the percentages of each subset measured by flow cytometry.

### Flow Cytometry

PBMC samples were analyzed using a FACSCanto flow cytometer (BD Biosciences) and FlowJo software (Tree Star, Ashland, OR, USA). Whole blood samples for counting absolute cell numbers were acquired and analyzed using MultiSet software in a FACSCalibur flow cytometer (BD Biosciences).

### Statistical Analysis

The results were analyzed using SPSS 13.0 (SPSS Inc. and Predictive Analytics, Chicago, IL, USA). Comparisons between healthy volunteers and patients were performed using the Mann-Whitney test; comparisons between patients’ samples (D7 versus D0) were performed using the Wilcoxon Signed-Rank test. *P*≤0.05 was considered statistically significant.

## Results

### Epidemiology of the Cohort Studied

At the time of admission, of the 67 patients enrolled in the study, 3% had sepsis, 17.9% had severe sepsis, and 79.1% had septic shock. The mean age was 63.1±17.3 years, and 62.7% were male. The primary sources of infection were the lung (41.8%), abdomen (25.4%), urinary tract (13.4%), bloodstream (6.0%), skin or soft tissue (4.5%), operative wound (3%), endocarditis (3%) and others (1.5%). The mean Acute Physiology and Chronic Health Evaluation II (APACHE II) score at enrollment was 18.6, ranging from 6 to 35, and the mean Sequential Organ Failure Assessment (SOFA) score was 7.6, ranging from 0 to 14. The in-hospital mortality was 38.8%. The mean age of healthy volunteers was 59.6±16.4 years, and 62.5% were male.

### Th1, Th17 and Treg Subsets in Septic Patients

The percentages of T helper (CD3+CD8−) lymphocytes spontaneously producing IFN-γ and IL-17 were higher in septic patients at enrollment (D0) than in healthy volunteers (Table 1). After PMA/Io stimulation, the percentage of T helper (CD3+CD8−) lymphocytes producing IFN-γ was lower, and the percentage producing IL-17 was higher in septic patients than in healthy volunteers (Table 1). No differences in the percentages of Treg cells were found between the two groups (Table 1).

**Table 1:** Percentages of T helper (CD3+CD8−) lymphocytes producing IFN-γ and IL-17, T helper (CD3+CD4+) expressing CD127-Foxp3+ and monocytes (CD14 positive) expressing CD163 or CD206 in healthy volunteers and septic patients (D0 samples).

		Healthy volunteers		Septic patients		
		%	n	%	n	*P* values
**T helper lymphocytes**						
IFN-γ	Control	0.07	30	0.12	59	0.020
		(0.05–0.09)		(0.05–0.21)		
	PMA/Io	21.40	30	10.40	59	0.001
		(14.48–27.03)		(7.15–20.20)		
IL-17	Control	0.11	30	0.21	59	<0.001
		(0.07–0.15)		(0.12–0.37)		
	PMA/Io	0.85	30	1.14	59	0.027
		(0.65–1.31)		(0.79–2.05)		
Foxp3+CD127−		3.11	32	2.40	62	0.216
		(1.99–5.40)		(1.09–4.61)		
**Monocytes**						
CD163		0.00	32	23.73	62	<0.001
		(0.00–0.86)		(3.85–39.46)		
CD206		0.48	32	2.38	62	<0.001
		(0.00–1.00)		(1.03–5.61)		

Values represent the median and 25–75% quartile. n  =  number of individuals in each group. The Mann-Whitney test was applied.

The absolute counts of CD4+ T lymphocytes in whole blood dramatically decreased in D0 samples from septic patients compared with healthy volunteers. Thus, the absolute numbers of T helper (CD3+CD8−) lymphocytes producing PMA/Io-induced IFN-γ and IL-17 and expressing Foxp3 (Treg) were lower in septic patients than in the controls (Table 2).

**Table 2:** Absolute counts of CD4+ T lymphocytes and subpopulations (CD3+CD8−) producing IFN-γ and IL-17 after PMA/Io stimulation, and T helper (CD3+CD4+) expressing CD127-Foxp3+ in healthy volunteers (n = 30) and septic patients (D0 samples, n = 46).

	Healthy volunteers	Septic patients	
	cell/µ L	cell/µ L	*P* values
**Lymphocytes**			
CD4+ T	972.50	342.00	<0.001
	(781.00–1165.75)	(159.50–577.50)	
IFN-γ	166.00	35.22	<0.001
	(124.89–283.58)	(14.79–98.86)	
IL-17	7.93	3.35	<0.001
	(5.93–12.63)	(1.80–6.47)	
Foxp3+CD127−	28.19	7.47	<0.001
	(16.20–49.11)	(3.29–13.50)	

Values represent the median and 25–75% quartile. The Mann-Whitney test was applied.

### T helper Subsets in Patients’ follow Up Samples

**Figure 4:**
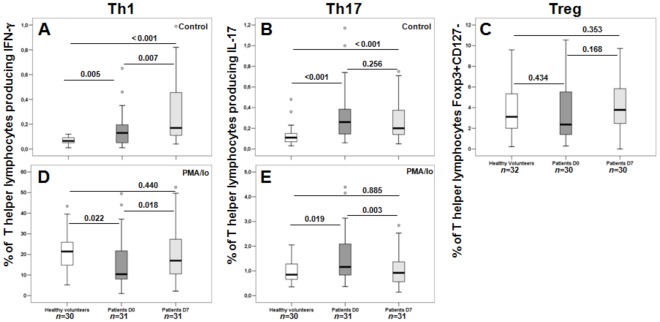
Percentages of T helper (CD3+CD8−) producing IFN-γ (Th1 - A/D) and IL-17 (Th17 - B/E), and percentages of Treg (CD3+CD4+CD127-Foxp3+ - C) lymphocytes in healthy volunteers and septic patients with paired samples (D0 and D7). n  =  number of individuals in each group. *P* values are shown. The Mann-Whitney test was used to compare healthy volunteer and patient samples (D0 and D7), and the Wilcoxon Signed-Rank Test was used to compare D0- and D7-related samples.

A spontaneous increased percentage of T helper (CD3+CD8−) lymphocytes producing IFN-γ and IL-17 was observed in patients’ D0 and D7 samples compared with healthy individuals (Figures 4A and B). Septic patients showed a dichotomous response after stimulation with PMA/Io; they presented a lower percentage of IFN-γ-producing cells (Figure 4D) and an increased percentage of IL-17-producing cells (Figure 4E) in admission samples (D0) compared with healthy individuals. Thus, admission samples in the group of septic patients with paired D0 and D7 samples presented results similar to the whole cohort of patients. In follow-up samples, a higher percentage of IFN-γ and a lower percentage of IL-17-producing cells were observed in D7 samples compared with D0 samples. No differences were observed between the samples from healthy volunteers and D7 samples for either cytokine analyzed (Figures 4D and E). Additionally, no difference was found in the percentage of regulatory T lymphocytes among the different groups (Figure 4C).

**Table 3:** Percentages of T helper lymphocytes producing IFN-γ, IL-17 (CD3+CD8−), T helper (CD3+CD4+) expressing CD127−Foxp3+ and monocytes (CD14 positive) expressing CD163 or CD206 in septic patients at admission (D0), divided into non-survivors (n = 24) and survivors (n = 35). Values represent the median and 25–75% quartile.

		Non-survivors	Survivors	
		%	%	*P* values
**T helper lymphocytes**				
IFN-γ	Control	0.16	0.12	0.588
		(0.05–0.26)	(0.06–0.20)	
	PMA/Io	10.03	12.8	0.294
		(4.56–21.30)	(8.04–18.60)	
IL-17	Control	0.29	0.19	0.081
		(0.16–0.42)	(0.12–0.34)	
	PMA/Io	1.10	1.14	0.871
		(0.81–1.94)	(0.80–2.19)	
Foxp3+CD127−		2.49	2.40	0.251
		(1.51–5.94)	(0.90–4.29)	
**Monocytes**				
CD163		26.63	23.51	0.378
		(6.29–44.32)	(3.77–39.22)	
CD206		4.33	2.02	0.054
		(1.56–6.99)	(1.06–3.71)	

The Mann-Whitney test was applied.

### T helper Subsets and Patients’ Outcomes

**Figure 5:**
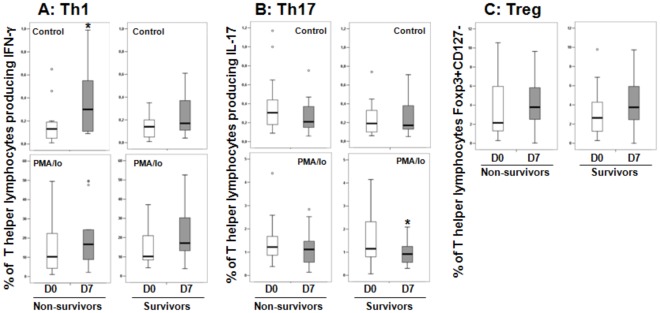
Percentages of T helper (CD3+CD8−) producing IFN-γ (Th1 - A/D) and IL-17 (Th17 - B/E), and percentages of Treg (CD3+CD4+CD127Foxp3+ - C) lymphocytes in non-survivors (n = 14) and survivors (n = 17) at admission (D0) and after 7 days (D7) in patients with follow-up samples. *P<0.05 (Wilcoxon Signed-Rank Test).

The percentages of Th1, Th17 and Treg cells were analyzed in relation to the patients’ in-hospital mortality. No differences were observed between the percentages of the T helper (CD3+CD8+) lymphocyte subsets at D0 between the survivors and the non-survivors (Table 3). In follow-up samples, a higher percentage of cells spontaneously producing IFN-γ was found in D7 samples compared with D0 samples from patients who had died (Figure 5A). A decreased percentage of PMA/Io-induced IL-17-producing cells was found in the survivors’ follow-up samples (D7) compared with the admission samples, while the percentage remained similar in non-survivors (Figure 5B). No changes in the levels of Tregs were found between D7 and D0 samples that correlated with clinical outcomes (Figure 5C).

### Septic Patients showed Increased Expression of CD163 and CD206 on Monocytes

A striking difference in the percentage of monocytes expressing CD163 or CD206 was found between healthy volunteers and septic patients, with the highest values observed in septic patients (Table 1).

In patients with sepsis, the percentages of cells expressing CD163 was higher in CD14++CD16− (median: 29.6%) and CD14++CD16+ (median: 27.00%) subsets than in the CD14+CD16+ subset (median: 0.00%). Similarly, the expression of CD206 was higher in CD14++CD16− (median: 2.83%) and CD14++CD16+ (median: 5.25%) than in the CD14+CD16+ subset (median: 0.00%). The expression of CD163 was higher in septic patients than in healthy volunteers in CD14++CD16− (29.18%, 25–75% quartile: 7.47–45.75% versus 0.00%, 25–75% quartile: 0.00–2.21, respectively; *P*<0.001), CD14++CD16+ (26.98%, 25–75% quartile: 3.82–40.92% versus 0.00%, 25–75% quartile: 0.00–0.00%, respectively; *P*<0.001) and CD14+CD16+ (0.00%, 25–75% quartile: 0.00–7.14% versus 0.00%, 25–75% quartile: 0.00–0.00%, respectively; *P*<0.001) monocytes. The expression of CD206 was higher in septic patients than in the controls in CD14++CD16− (2.83%, 25–75% quartile: 1.05–6.31% versus 0.83%, 25–75% quartile: 0.35–1.70%, respectively; *P*<0.001) and CD14++CD16+ (5.25%, 25–75% quartile: 0.81–1.19% versus 0.08%, 25–75% quartile: 0.00–4.19, respectively; *P*<0.001), and it was similar in CD14+CD16+ (0.00%, 25–75% quartile: 0.00–2.30% versus 0.00%, 25–75% quartile: 0.00–0.93%, respectively; *P* = 0.069) monocytes.

**Figure 6:**
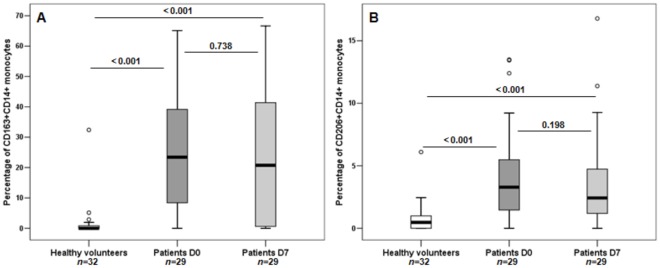
Percentages of AAM expressing CD163 (A) and CD206 (B) in healthy volunteers and septic patients with paired samples (D0 and D7). n =  number of individuals in each group. *P* values are shown. The Mann-Whitney test was used to compare healthy volunteer and patient samples (D0 and D7), and the Wilcoxon Signed-Rank Test was applied to patients’ related samples.

The percentage of monocytes expressing CD163 or CD206 remained high in patients’ follow-up samples, with similar results obtained in D7 samples compared with D0 samples; the percentages at both time points (D0 and D7) were higher than in healthy volunteers (Figures 6A and 6B).

### Expression of CD163 and CD206 on Monocytes and Patients’ Outcomes

Similar percentages of CD163-expressing monocytes were found in the survivors and non-survivors. Additionally, there were no significant differences in the percentages of CD206-positive monocytes between the survivors and non-survivors (Table 3). No differences were found between the D0 and D7 samples in surviving and non-surviving patients for either of the receptors analyzed (data not shown).

## Discussion

Our results show profound lymphopenia and decreased CD4+T cell counts in septic patients, as previously described [23], [33]. This low CD4+T cell number impacted all three of the subpopulations we evaluated, Th1, Th17 and Treg, which presented reduced absolute numbers. However, there was a diverse impact on the different subsets of these cells, as evidenced by their differential percentages in septic patients and healthy volunteers.

We found increased percentages of cells spontaneously producing IFN-γ or IL-17, which is consistent with the ongoing inflammatory process in septic patients. However, upon stimulation with PMA/Io, a decreased percentage of cells producing IFN-γ and an increased percentage of cells producing IL-17 were observed in admission samples from septic patients compared with healthy volunteers. The observation of a lower percentage of T helper lymphocytes producing IFN-γ is consistent with previous work showing a shift from Th1 to Th2 cytokine profiles following trauma, burns and infection. In agreement with this, O’Sullivan et al. [34] observed decreased production of IFN-γ and increased production of IL-4 in patients with burns and trauma, while Heidecke et al. [35] found decreased T lymphocyte production of IL-2, TNF-α and IFN-γ, the latter in early lethal sepsis, and unaffected production of IL-4 and IL-10 in patients with peritonitis. The increased percentage of IL-17-producing T helper lymphocytes in the peripheral blood of septic patients is consistent with the increased percentage of monocytes expressing CD206, which has been shown to be involved in triggering Th17 in response to *Candida albicans* [36], and with the unchanged percentage of Treg, which would have an inhibitory effect on Th17 differentiation.

Most of the literature regarding the role of Th17 in disease comes from studies using autoimmune models [6], yet there is increasing evidence for a central role of Th17 in infections. The IL-17 receptor (IL-17R) has a protective role in a model of *Klebsiella pneumoniae* lung infection in mice [37]. Impaired neutrophil mobilization and influx into infected organs were also found in IL-17R(−/−) mice infected with *Candida albicans*, whereas the expression of IL-17A protected wild-type mice from a lethal challenge [38]. In contrast, Flierl et al. showed that the intravenous injection of anti-IL-17 improved survival in mice subjected to cecal ligation and puncture (CLP)-induced sepsis and decreased the serum levels of IL-6 and TNF-α [39]. Th17 cells are a source of IL-21 and IL-22, in addition to IL-17A and IL-17F. In a recent report, increased IL-22 plasma levels were found in patients with abdominal sepsis [40]. Our finding of an increased proportion of IL-17-producing T helper lymphocytes in septic patients (D0) suggests a shift in the differentiation of T helper lymphocytes toward an inflammatory pattern that will, in turn, drive innate immune cells toward activation. Previous work from our group and from others support the notion that there is a state of neutrophil activation in septic patients, at least with respect to phagocytosis, generation of reactive oxygen species [27], [41] and TLR pathway gene expression [42].

We found reduced Treg cell absolute numbers in septic patients at the time of admission, reflecting the decreased numbers of CD4+T cells, but the percentage of Tregs remained unchanged between patients and healthy volunteers. In a pioneering report, Monneret et al. found an increased percentage of TCD4+CD25+ cells in septic patients [8], due to a decreased proportion of TCD4+CD25− cells [9]. On the other hand, Hein et al. found similar proportion of Tregs in admission samples from septic patients compared with healthy volunteers [13]. Our results further support that the reported increase in the percentages of Tregs among T helper cells in septic patients is inconsistent across all septic patient populations.

In contrast to our results and previous ones showing modulation of Th cell sub-populations in septic patients, Venet and coworkers [43] recently showed that all CD4+T lymphocyte subsets were equally diminished in septic shock patients. They evaluated CD4+ T cell-specific transcription factors for Th1 (T-bet), Th2 (GATA-3), Treg (Foxp3) and Th17 (RORγT) in septic patients during the first 48 h of shock. Possible explanations for the discrepancy among the studies may be the variations in the approaches used for collecting the samples and for characterizing T helper sub-populations.

In the follow-up samples the percentage of IFN-γ-producing T helper lymphocytes upon PMA/Io after 7 days of therapy was higher than at admission, whereas the percentage of IL-17-producing T helper lymphocytes was lower. Thus, the proportions of both sub-populations after 7 days of therapy were similar to those in healthy volunteers, reversing the Th1 down-regulation and Th17 up-regulation observed at admission. This may indicate an effort to restore homeostasis during therapy. Interestingly, while the percentages of Th1 and Th17 at admission (D0) did not correlate with in-hospital survival rates, the percentages of IFN-γ-spontaneously producing T helper cells were higher at day 7 than at admission in the non-survivors, and the percentage of PMA/Io-induced IL-17 cells was lower at day 7 than at admission in the survivors. These results suggest that the sustained state of Th1 and Th17 activation may be deleterious in septic patients. Again, no difference in the percentage of Tregs was found during patient follow-up when compared with D0.

To further evaluate the mononuclear cell subpopulations in septic patients, we measured the expression of markers for alternatively activated monocytes. We found a dramatic increase in the percentage of monocytes expressing CD163 and CD206 in septic patients. CD163 expression on neutrophils and monocytes has been previously evaluated in critically ill neonates and children. It was shown that the CD163 mean fluorescence intensity for neutrophils and monocytes and the CD163 index for neutrophils could be used to discriminate between SIRS and sepsis in this population [44]. sCD163, the soluble form of CD163, has been evaluated as a serum marker for bacteremia, and its levels have been shown to be correlated with clinical outcomes [45], [46]. In our study, there was no association between the cell surface expression of CD163 and clinical outcomes, but the striking up-regulation of CD163 suggests it may be a good diagnostic marker. Future studies with critically ill non-septic patients are needed to address this issue. In contrast, there was a trend toward higher percentages of monocytes expressing CD206 in the non-surviving patients. However, the small number of patients in the survival and non-survival groups necessitates further studies for the evaluation of its prognostic value.

Human and mice monocytes have also been characterized as classical and non-classical or inflammatory and resident monocytes based on CD14/CD16 (for humans) and Ly6C/CX3CR1/CCR2 (for mice) expression [47]–[49]. The expression of CD16 is increased in M1 monocytes and the expression of CD14, CD206 and CD163 is increased in M2 monocytes [50]. In a study evaluating HIV-infected individuals and healthy volunteers, Tippett et al. reported that the expression of CD163 was highest in CD14++CD16−, intermediate in CD14++CD16+ and lowest in CD14+CD16++ monocyte subsets. CD163 expression was higher in HIV-infected individuals than in controls only in the CD14++CD16+ subset [51]. When analyzing our data on the monocyte subsets CD14++CD16−, CD14++CD16+ and CD14+CD16+, we found higher expression of CD163 and CD206 in septic patients in the CD14++CD16− and CD14++CD16+ subsets than in the CD14+CD16+ subset. Additionally, the expression levels of CD163 and CD206 were higher in septic patients than in controls in all monocyte subsets, except for CD206 in CD14+CD16+.

The increased proportion of monocytes expressing markers for alternatively activated monocytes/macrophages found in our study may result from a lower proportion of IFN-γ-producing T helper cells, which induce the classically activated macrophages, or from the previously described increased Th2 profile found in septic patients. CD4+CD25+Foxp3+ regulatory T cells also induce alternatively activated monocytes, with up-regulation of both receptors on monocytes [22], yet we found similar percentages of Tregs in patients and healthy volunteers. IL-21, a cytokine linked to the Th17 profile, induces Th2 effector functions and alternative macrophage activation [21], and conversely, the mannose receptor has been shown to be crucial for the induction of IL-17 by *Candida albicans* [36]. Thus, the increased expression of CD206 may be related to the increased Th17 we found in septic patients. However, despite the clear interplay between monocytes and T helper lymphocytes in the differentiation of subpopulations, we did not find a correlation between T helper lymphocyte subpopulations and expression of markers for alternatively activated monocytes (data not shown). We are currently evaluating the levels of circulating cytokines related to Th1, Th2, Treg and Th17 profiles in septic patients to help understand the modulation of mononuclear subsets in septic patients.

It has been well documented that monocytes from septic patients fail to produce TNF-α and IL-6 [3], [4], while the production of IL-10 seems to be less affected [4], [24]. Previous studies by our group also showed that monocytes from patients with severe sepsis and septic shock produce low amounts of inflammatory cytokines while preserving the ability to generate reactive oxygen species (ROS) [24], [27]. Supporting these results, monocytes that were tolerant to LPS *in vitro* produced low amounts of IL-6, showed preserved phagocytosis and increased levels of ROS [52]. Altogether, the above results clearly indicate a reprogramming of monocyte/macrophage function during sepsis and LPS-induced tolerance [28], [53]. This phenotype may correspond, at least in part, to alternative monocyte activation. The increased expression of CD206 and CD163 on circulating monocytes in our study supports the conclusion that alternative activation of monocytes occurs during sepsis. Future studies combining cell surface expression and functional evaluation of circulating monocytes may provide relevant information about the different phenotypes of monocytes during sepsis, which would account for a broad spectrum of monocyte differentiation or activation.

There are some limitations to this study. Because no specific marker was used to exclude NKT cells from the CD3+CD8− gated cells, we should consider that these cells may also contribute to the IFN-γ and IL-17-producing cells. Without evaluating the Th2 subpopulation, our analysis of mechanisms inducing monocyte differentiation toward an M2 profile is incomplete. Additionally, while the use of flow cytometry for the detection of intracellular cytokines allows for the functional characterization of T lymphocyte subtypes at the cellular level, *in vitro* stimulation may lead to cell division or death that may differ between healthy volunteers and septic patients. To address this in the future, a viability marker could be used to exclude dead or early stage apoptosis cells in the flow cytometry analysis. As with other studies, the evaluation of peripheral blood cell function in septic patients may not correspond to the processes in other organs and tissues. We did not perform functional studies of monocytes, which would add relevant information. Nevertheless, the expression of CD206 and CD163 on monocytes of septic patients clearly indicates a pattern of cell differentiation.

In conclusion, patients with sepsis presented an increased percentage of Th17 and a decreased percentage of Th1 cells upon treatment with PMA/Io, and these changes were reversed after 7 days of therapy, while no changes were found in percentages of the Treg subset. We found a dramatic increase in the percentage of monocytes expressing CD206 and CD163, indicating the alternative activation of monocytes, which supports previous studies showing a reprogramming of monocyte function during sepsis.
